# Supplementary material for: RNA-binding proteins Musashi and tau soluble aggregates initiate nuclear dysfunction
Source: Nat Commun. 2020 Aug 27;11:4305. doi: 10.1038/s41467-020-18022-6 (PMC7453003; doi:10.1038/s41467-020-18022-6)
Supplement: Supplementary file 1 — Supplementary Information [file 41467_2020_18022_MOESM1_ESM.pdf]

Supplementary Information

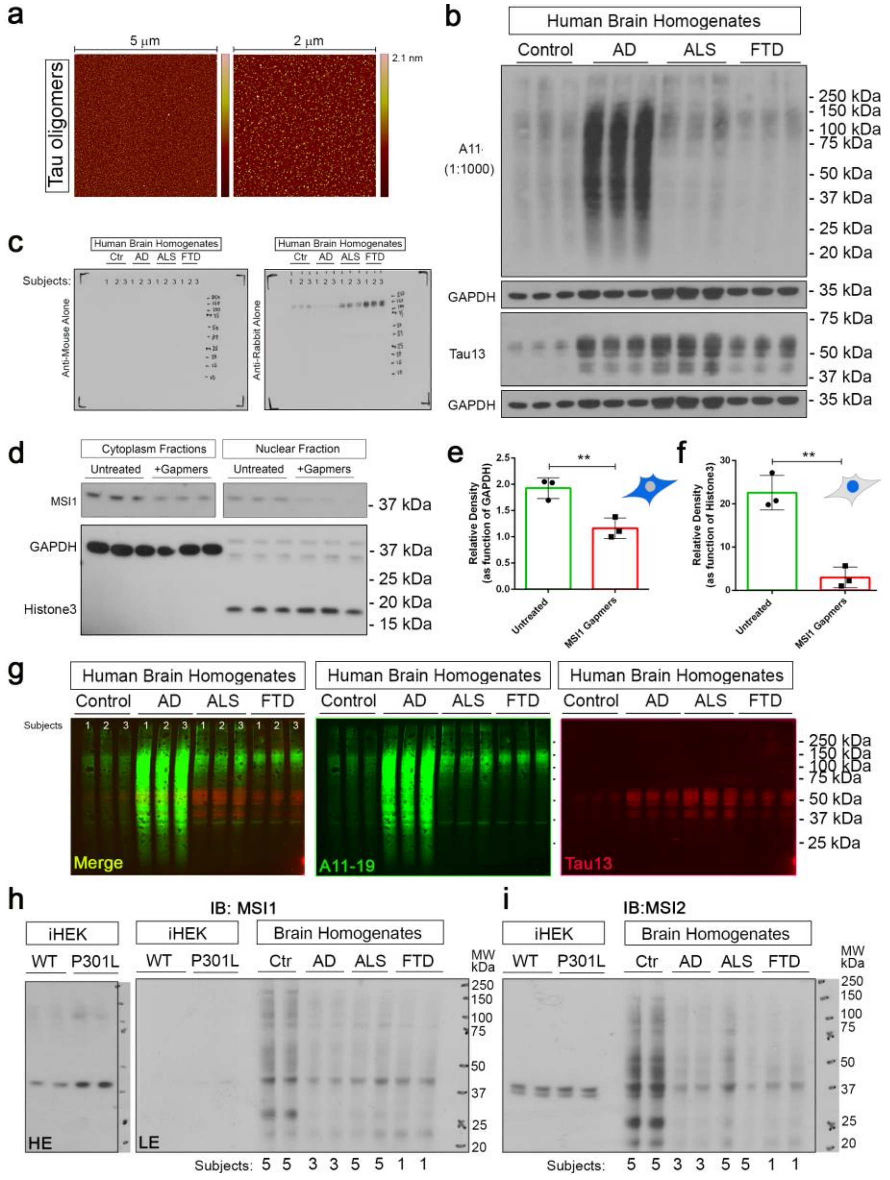

Supplementary Figure 1. (**a**) Representative Images of TauO with AFM (5µm and 2µm size fields). (**b**) WB of A11 and Tau13 antibodies in control, AD, ALS and FTD frontal cortex brain homogenates. (**c**) Human frontal cortex brain homogenates membranes with anti-mouse and anti-rabbit secondary alone. (**d**) IB of MSI1, GAPDH and Histone3 in cytoplasm and nuclear fractions of P301L tau iHEK transfected with MSI1 gapmers. (**e**) Relative density of cytoplasmic MSI1 (UT vs. Gapmers p=0.0091**, two-tailed unpaired t-test). N=3 biologically independent cells examined over three independent experiments. Data are presented as mean ± SD. (**f**) Relative density of nuclear MSI1 (UT vs. Gapmers p=0.0019**, two-tailed unpaired t-test). N=3 biologically independent cells (ROIs) examined over three independent experiments. Data are presented as mean ± SD. (**g**) Li-Cor fluorescent IB images of A11 (green) and Tau13 (red) antibodies in human frontal cortex brain homogenates membrane, single channels and merge images are represented. (**h, i**) MSI1 and MSI2 IB in WT/P301L tau iHEK (total lysates) and human brain homogenates. Source data are provided as a Source Data file.

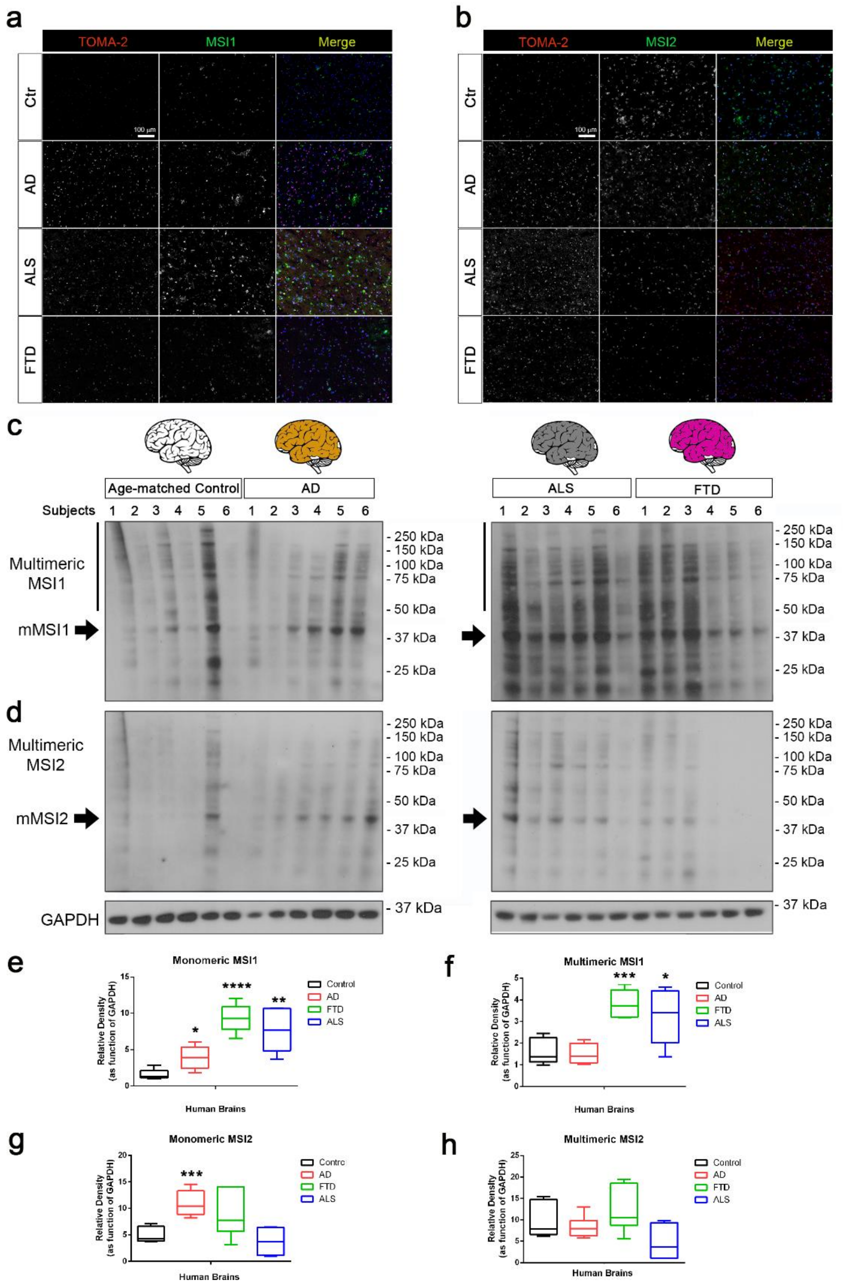

Supplementary Figure 2. (**a**) Representative co-immunofluorescence of MSI1/TOMA-2 in control, AD, ALS and FTD brains. (**b**) Representative co-immunofluorescence of MSI2/TOMA-2 in control, AD, ALS and FTD brains. All slides are stained with DAPI (Nuclei). Magnification 10x (white scale bar: 100µm). (**c**) MSI1 IB in human brain homogenates: age-matched controls (6 cases), AD (6), ALS (6) and FTD (6). (**d**) MSI2 IB in human brain homogenates: age-matched controls (6 cases), AD (6), ALS (6) and FTD (6). (**e**) Monomeric MSI1 relative density (as function of GAPDH) in control and diseased human brains (two-tailed t-test; Ctr vs. AD *p=0.0139**, Ctr vs. FTD *p<0.0001*****, Ctr vs. ALS *p=0.0014***). Data presented as box-and-whisker plots. Variability is shown using medians (line in the box), 25th and 75th percentiles (box), and min to max (whiskers). (**f**) Multimeric MSI1 relative density (as function of GAPDH) in control and diseased human brains (two-tailed t-test; Ctr vs. AD *p=0.724; ns*, Ctr vs. FTD *p=0.0002****, Ctr vs. ALS *p=0.0354**). Data presented as box-and-whisker plots. Variability is shown using medians

(line in the box), 25th and 75th percentiles (box), and min to max (whiskers). (**g**) Monomeric MSI2 relative density (as function of GAPDH) in control and diseased human brains (two-tailed t-test; Ctr vs. AD *p=0.0010****, Ctr vs. FTD *p=0.3559; ns*, Ctr vs. ALS *p=0.0963; ns*). Data presented as box-and-whisker plots. Variability is shown using medians (line in the box), 25th and 75th percentiles (box), and min to max (whiskers). (**h**) Multimeric MSI2 relative density (as function of GAPDH) in control and diseased human brains (two-tailed t-test; Ctr vs. AD *p=0.4743; ns*, Ctr vs. FTD *p=0.0591, ns*, Ctr vs. ALS *p=0.3712; ns*). Data presented as box-and-whisker plots. Variability is shown using medians (line in the box), 25th and 75th percentiles (box), and min to max (whiskers). N=6 biologically independent samples examined over three independent WB experiments. Source data are provided as a Source Data file.

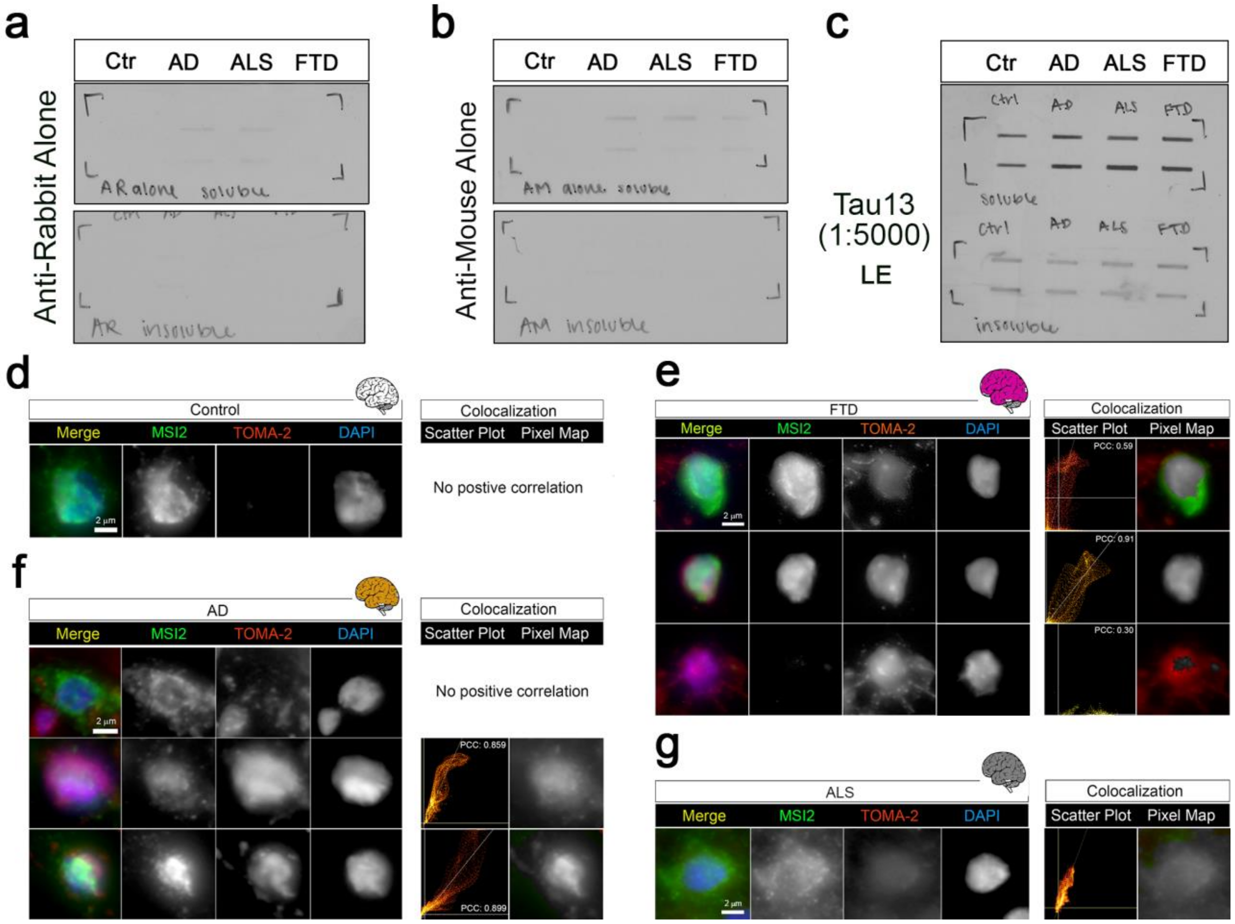

Supplementary Figure 3. (**a-c**) Filter trap assay of anti-Rabbit secondary, anti-Mouse secondary and Tau13 (1:5000) Antibodies, respectively, in Control, AD, ALS and FTD soluble and insoluble fractions of human brains. (**d**) Representative Co-IF of control brain cells stained with MSI2 (green) / TOMA-2 (red). (**e**) Representative Co-IF of FTD brain cells stained with MSI2 (green) / TOMA-2 (red). (**f**) Representative Co-IF of AD brain cells stained with MSI2 (green) / TOMA-2 (red). (**g**) Representative Co-IF of ALS brain cells stained with MSI2 (green) / TOMA-2 (red). All Co-IF present single channels (grey) and overlap: MSI2 (green) and TOMA-2 (red). For positive PCC relation Scatter Plots and Co-localization maps are presented for the relative image (white scale bar: 2µm). Source data are provided as a Source Data file.

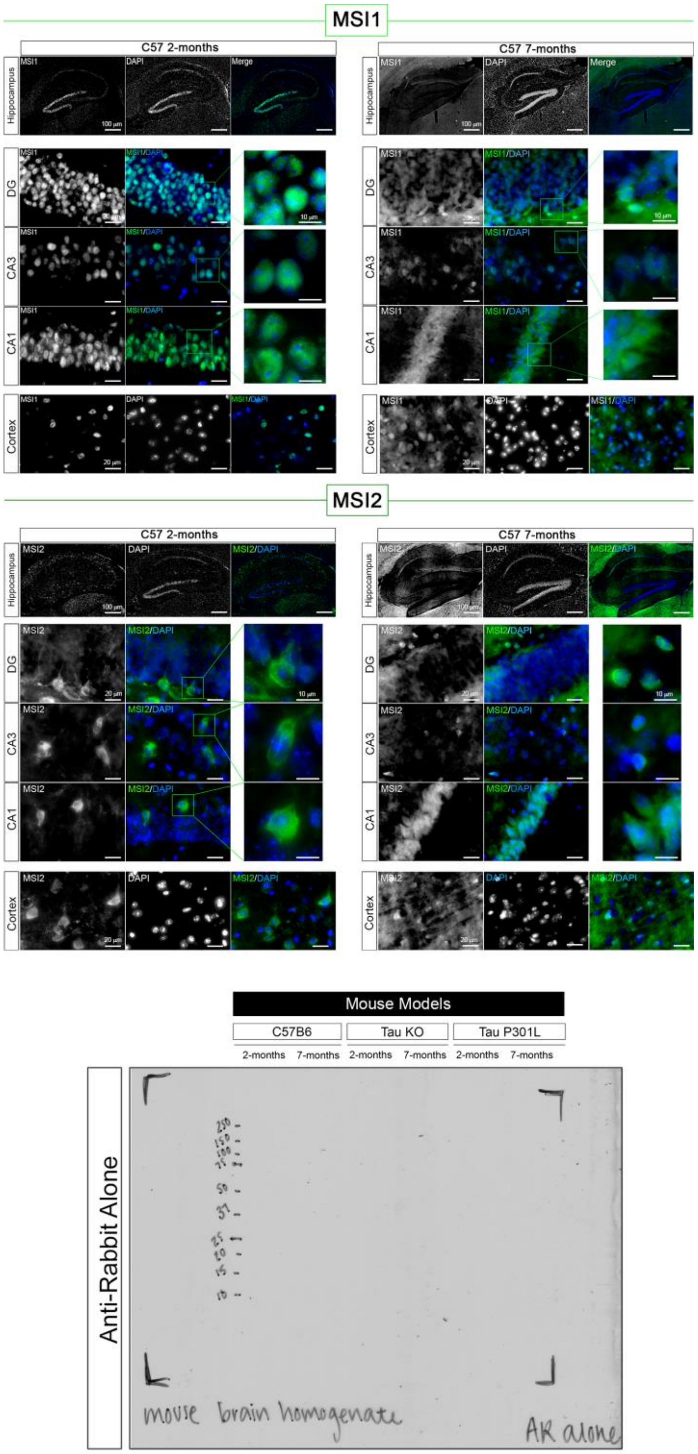

Supplementary Figure 4. Representative immunofluorescence of MSI1 (top panel) and MSI2 (bottom panel) in 2- and 7-month old C57BL/6 mice. Anti-Rabbit alone membrane of mouse brain homogenates. Source data are provided as a Source Data file.

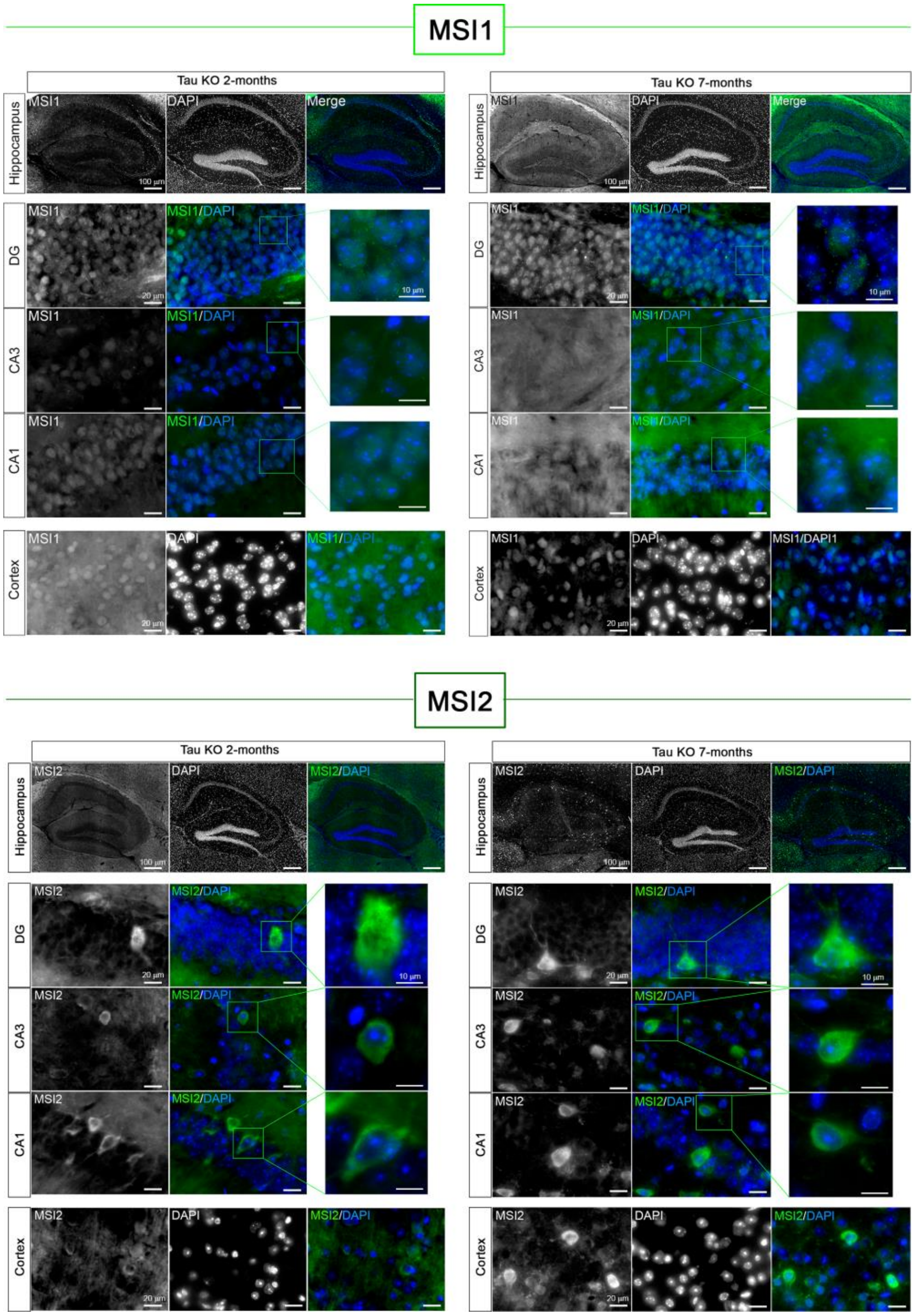

Supplementary Figure 5. Representative immunofluorescence of MSI1 (top panel) and MSI2 (bottom panel) in 2- and 7-month tau KO mice.

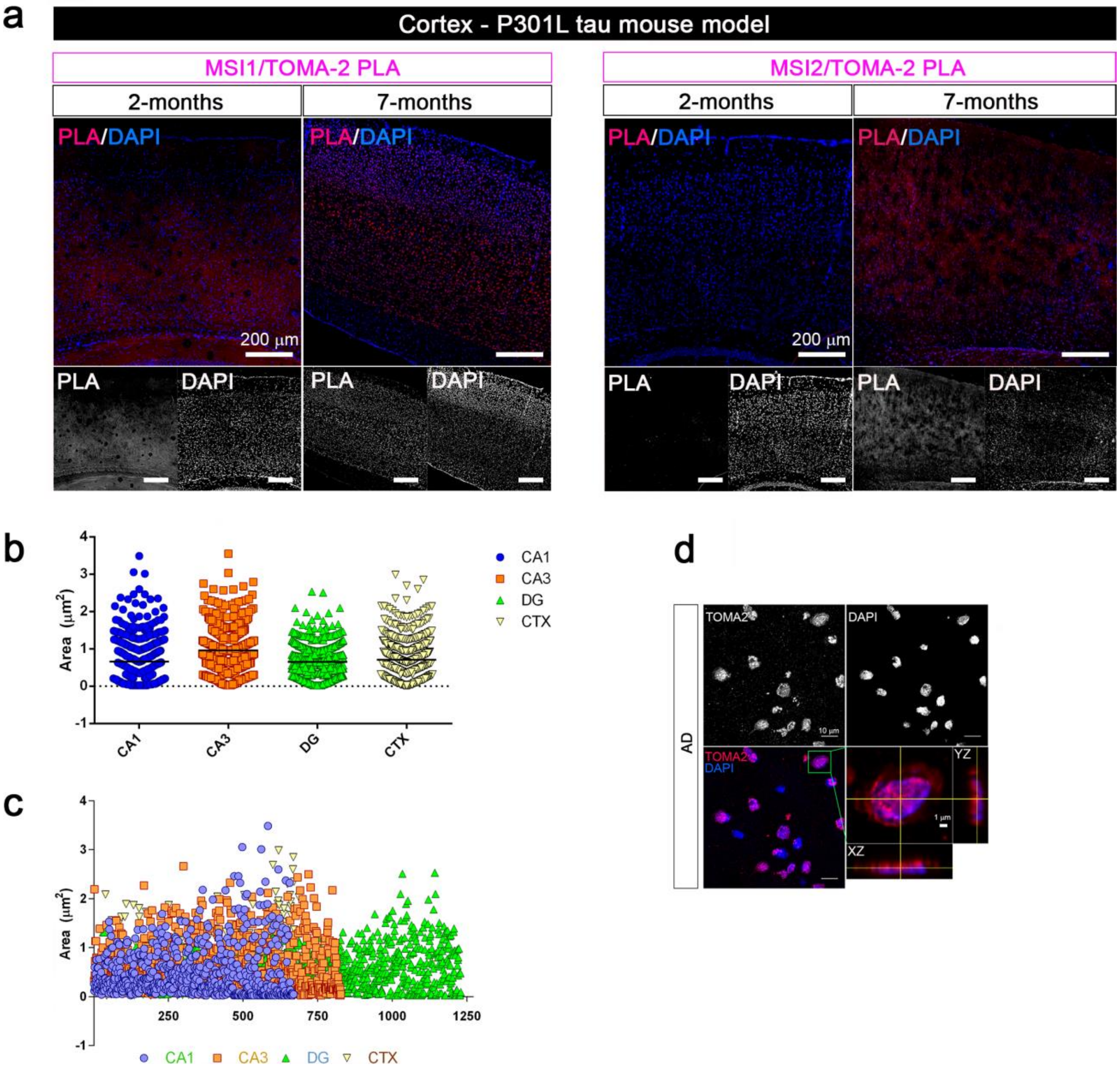

Supplementary Figure 6. (**a**) Representative PLA staining MSI1/TOMA-2 (left panel) and MSI2/TOMA-2 (right panel). PLA and DAPI channels are presented in grey on the bottom of each colored overlap. (**b**) Quantification of speckles area (MSI1/TOMA-2) in CA1, DG, CA3 and CTX regions. (**c**) Graph of Area distribution of speckles showed in B. (**d**) Representative confocal image of AD cortex stained with TOMA-2 (red) and DAPI (blue), TOMA-2 and DAPI are presented in gray with the merge and the orthogonal view. For single frames white scale bar: 10µm. Orthogonal view (bottom right) of enlarged ROI (green square) from the merge (white scale bar: 1µm). Source data are provided as a Source Data file.

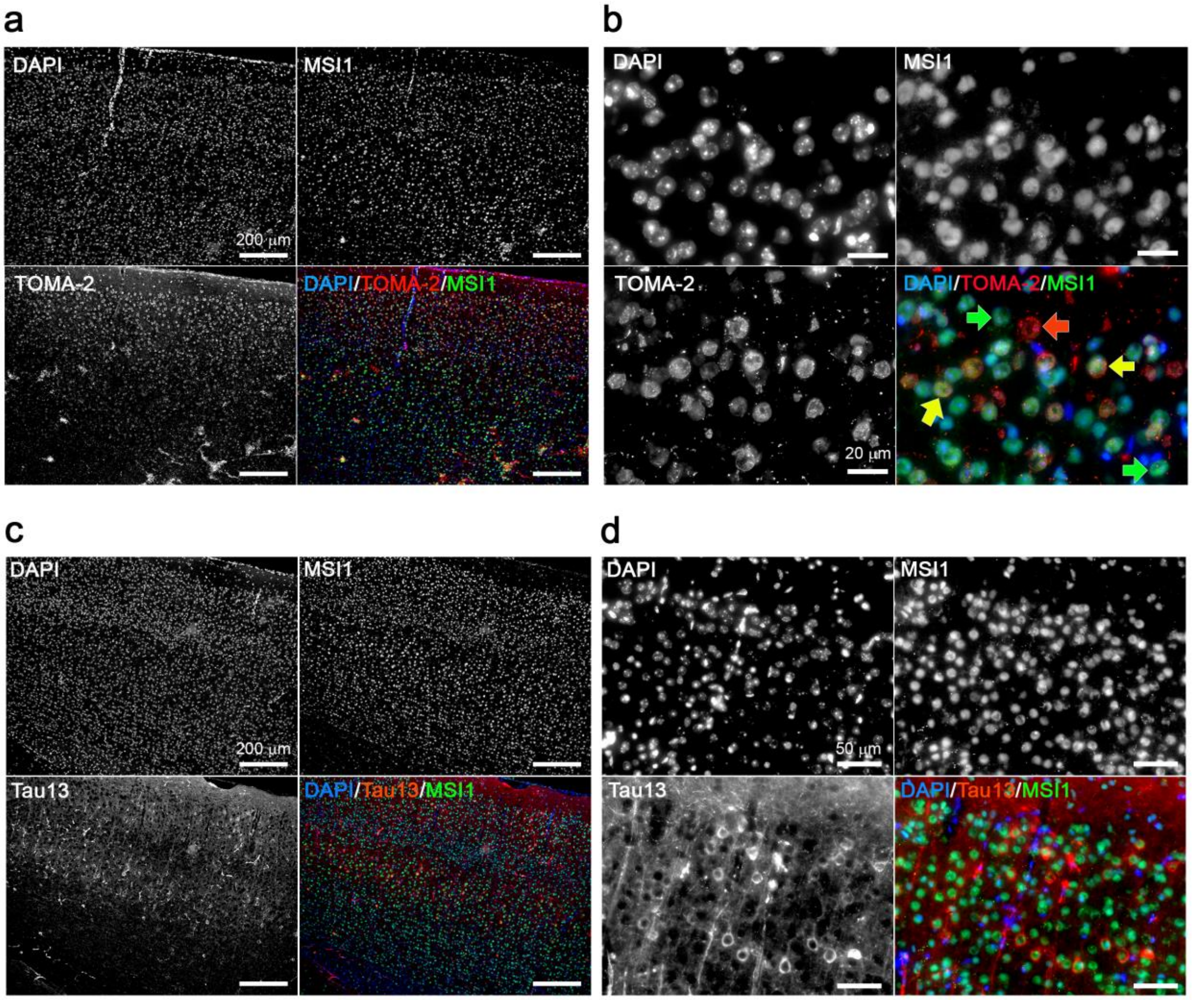

Supplementary Figure 7. (**a**) Representative IF of MSI1 (green), TOMA-2 (red) and Nuclei (DAPI, Blue) in P301L cortex 7-month old (Magnification 10X, white scale bar: 200µm). (**b**) Representative IF of MSI1 (green), TOMA-2 (red) and Nuclei (DAPI, Blue) in P301L cortex 7-month old (Magnification 100X, optical zoom 2X, white scale bar: 20µm). (**c**) Representative IF of MSI1 (green), Tau13 (red) and Nuclei (DAPI, Blue) in P301L cortex 7-month old (Magnification 10X, white scale bar: 200µm). (**d**) Representative IF of MSI1 (green), Tau13 (red) and Nuclei (DAPI, Blue) in P301L cortex 7-month old (Magnification 40X, white scale bar: 50µm).

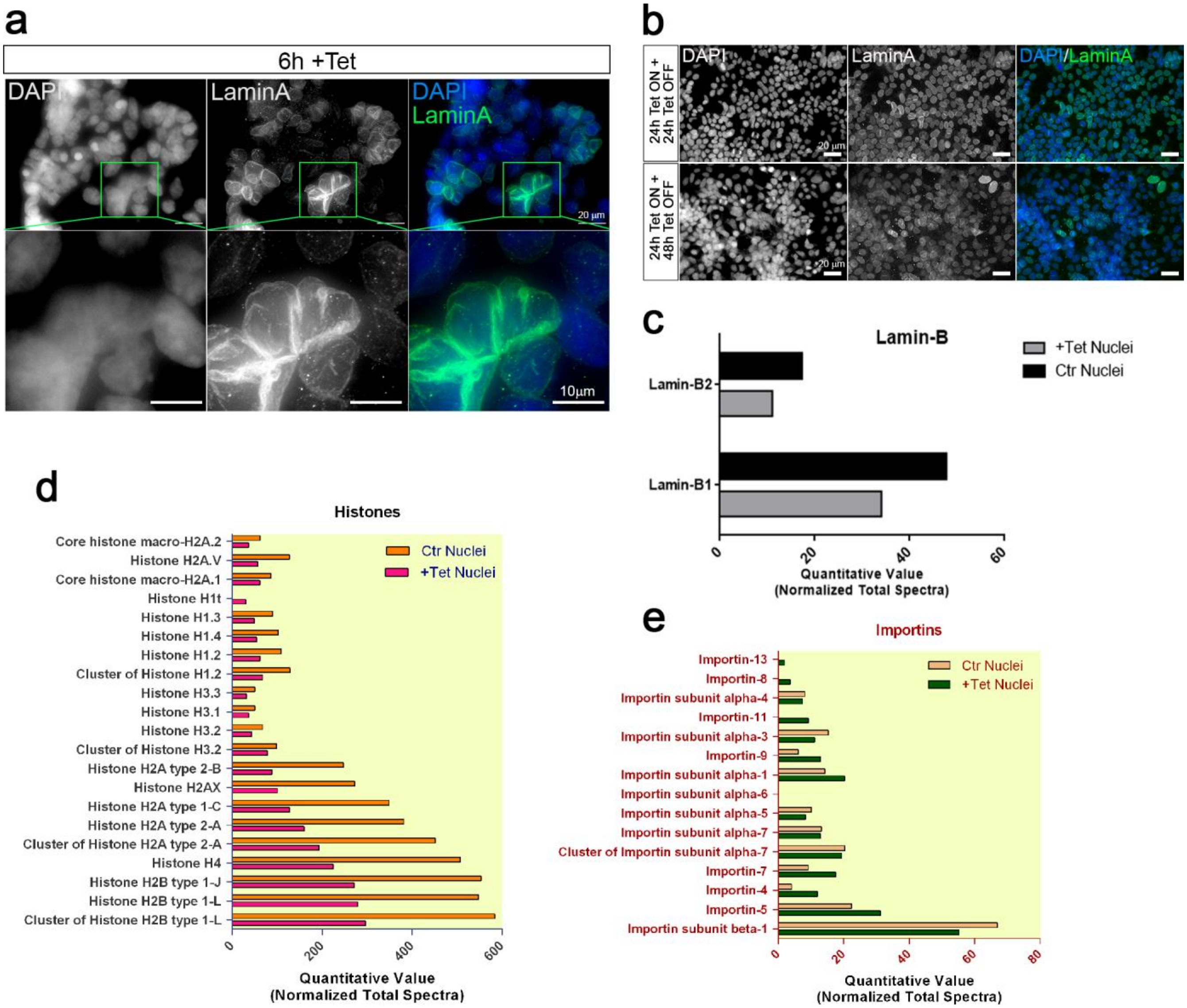

Supplementary Figure 8. (**a**) Representative LaminA (green) IF in P301L tau iHEK after 6h with Tet. Top Panel magnification: 20x (scale bar: 20µm). Bottom panel zoomed images from green boxes (white scale bar: 10µm). (**b**) Representative LaminA (green) IF in P301L tau iHEK after 24h Tet ON + 24h Tet OFF and 24h Tet ON + 48h Tet OFF (magnification: 20x). In all IF, nuclei are stained with DAPI (Blue). White scale bar: 20µm. (**c**) LaminB1 and LaminB2 levels by MS in Ctr and +Tet P301L tau iHEK. (**d**) Histones levels by MS in Ctr and +Tet P301L tau iHEK nuclear fractions. (**e**) Importins levels by MS in Ctr and +Tet P301L tau iHEK nuclear fractions. Source data are provided as a Source Data file.

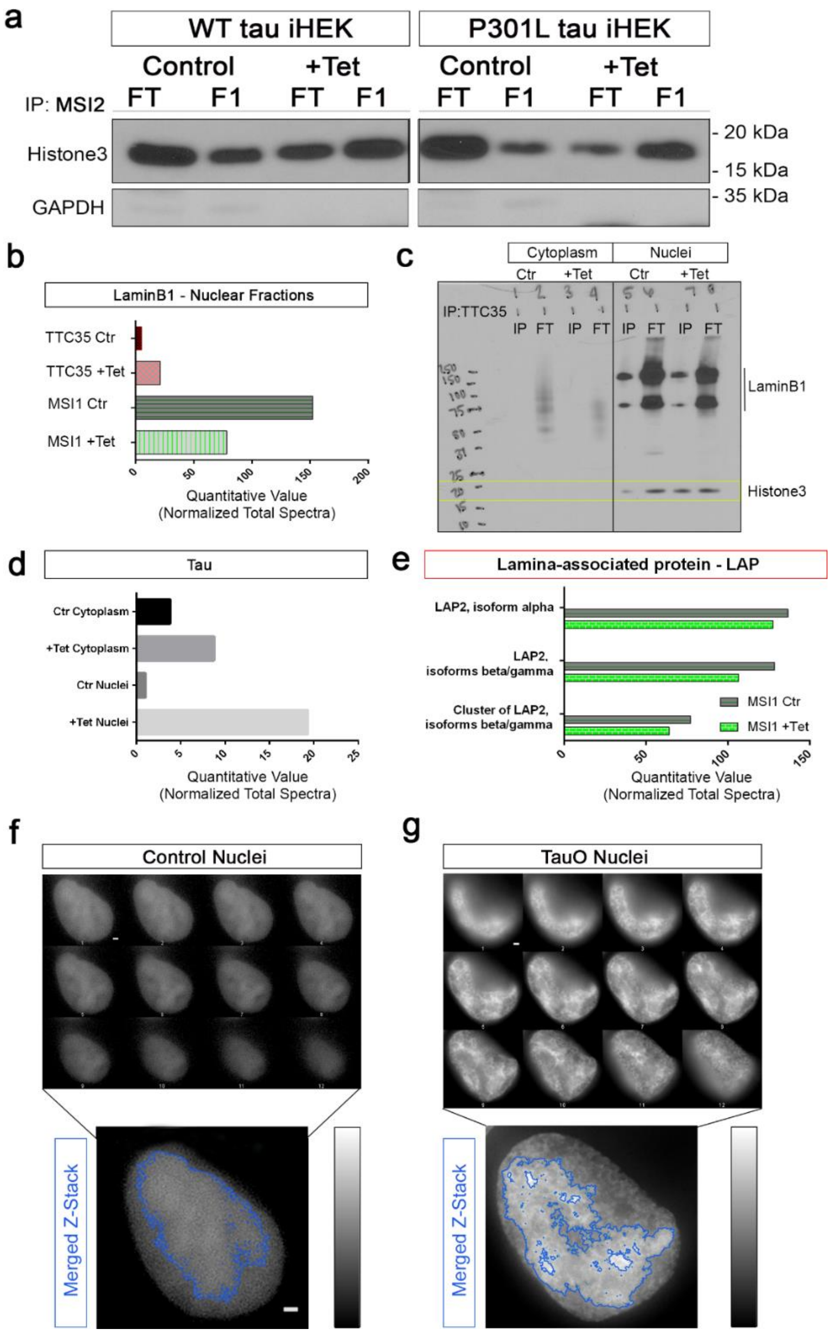

Supplementary Figure 9. (**a**) IB of Histone3 and GAPDH in IP MSI2 nuclear fractions of WT and P301L tau iHEK. (**b**) MS LaminB1 level in TTC35 and MSI1 IP nuclear fractions (Ctr and +Tet). IB of Histone3 in IP MSI1 cytoplasmic and nuclear fractions of P301L tau iHEK. (**c**) IB LaminB1 and Histone3 in TTC35 IP cytoplasm/nuclear fractions. (**d**) Tau level in P301L tau iHEK (cytoplasm and nuclear fractions) in Ctr and +Tet. (**e**) Lamina-Associated Proteins (LAPs) levels by MS in IP MSI1 Ctr vs +Tet (nuclear fractions). (**f**) Representative DAPI (grey) staining of untreated (Ctr) P301L tau iHEK nucleus. Nucleus is represented as full stack (bottom) and montage (top). (**g**) Representative nucleus DAPI (grey) staining of Tet induced P301L tau iHEK. Nucleus is represented as full stack (bottom) and montage (top). Grey color grade bar is presented. Source data are provided as a Source Data file.

Supplementary Table 1. List of RT-qPCR primers.

Human MAPT primers:	
Name: MAPT-2238F 25 bases<br>TM = 58C	Sequence: GCAGTGTGCAAATAGTCTACAAACC
Name: MAPT-2351R 22 bases<br>TM = 59C	Sequence: CAGATTTTACTTCCACCTGGCC<br>Amplicon = 114bp
