# Supplementary material for: RNA-binding proteins Musashi and tau soluble aggregates initiate nuclear dysfunction
Source: Nat Commun. 2020 Aug 27;11:4305. doi: 10.1038/s41467-020-18022-6 (PMC7453003; doi:10.1038/s41467-020-18022-6)
Supplement: Supplementary file 3 — Reporting summary [file 41467_2020_18022_MOESM3_ESM.pdf]

nature research

Corresponding author(s): Rakez Kayed

Last updated by author(s): Jul 2, 2020

Reporting Summary

Nature Research wishes to improve the reproducibility of the work that we publish. This form provides structure for consistency and transparency in reporting. For further information on Nature Research policies, see Authors & Referees and the Editorial Policy Checklist .

Statistics

For all statistical analyses, confirm that the following items are present in the figure legend, table legend, main text, or Methods section.

n/a	Confirmed	
☐	☒	The exact sample size (*n*) for each experimental group/condition, given as a discrete number and unit of measurement
☒	☐	A statement on whether measurements were taken from distinct samples or whether the same sample was measured repeatedly
☐	☒	The statistical test(s) used AND whether they are one- or two-sided<br>*Only common tests should be described solely by name; describe more complex techniques in the Methods section.*
☒	☐	A description of all covariates tested
☒	☐	A description of any assumptions or corrections, such as tests of normality and adjustment for multiple comparisons
☐	☒	A full description of the statistical parameters including central tendency (e.g. means) or other basic estimates (e.g. regression coefficient) AND variation (e.g. standard deviation) or associated estimates of uncertainty (e.g. confidence intervals)
☒	☐	For null hypothesis testing, the test statistic (e.g. *F*, *t*, *r*) with confidence intervals, effect sizes, degrees of freedom and *P* value noted<br>*Give P values as exact values whenever suitable.*
☒	☐	For Bayesian analysis, information on the choice of priors and Markov chain Monte Carlo settings
☒	☐	For hierarchical and complex designs, identification of the appropriate level for tests and full reporting of outcomes
☐	☒	Estimates of effect sizes (e.g. Cohen's *d*, Pearson's *r*), indicating how they were calculated

Our web collection on statistics for biologists contains articles on many of the points above.

Software and code

Policy information about availability of computer code

Data collection	BZ-X Viewer Keyence Microscope<br>BZ-X Wide Image Viewer Keyence Microscope<br>Microscope Software Black ZEN lite (Zeiss, UTMB Optical and Microscopy Core Facility)<br>XCalibur, version 2.1.0 (Thermo Fisher Scientific) (Mass-Spectroscopy Core Facility, UTMB)<br>UltiMate 3000 RSLCnano, Dionex (nano-LC chromatography system) (Mass-Spectroscopy Core Facility, UTMB)<br>Multimode 8 HR Atomic Force Microsocpy
Data analysis	GraphPad Prism 6 Graphpad.com (UTMB)<br>ImageJ FIJI ImageJ Free NIH<br>Analyzer BZ-X Keyence Microscope<br>Arivis Vision 4D – 3D Viewer Arivis AG www.arivis.com (UTMB Optical and Microscopy Core Facility)<br>Proteome Discoverer (Thermo Fisher, version 2.2.0388)<br>Uniprot Human database (version 06-27-2018)

For manuscripts utilizing custom algorithms or software that are central to the research but not yet described in published literature, software must be made available to editors/reviewers. We strongly encourage code deposition in a community repository (e.g. GitHub). See the Nature Research guidelines for submitting code & software for further information.

Data

Policy information about availability of data

All manuscripts must include a data availability statement. This statement should provide the following information, where applicable:

- Accession codes, unique identifiers, or web links for publicly available datasets
- A list of figures that have associated raw data
- A description of any restrictions on data availability

The original and analysed datasets generated during the current study, and the codes used to analyse them, are available from the corresponding author upon reasonable request. There is no restriction on material availability. We include a statement on data availability in our manuscript, as required by Nature policy.

Field-specific reporting

Please select the one below that is the best fit for your research. If you are not sure, read the appropriate sections before making your selection.

[x] Life sciences [ ] Behavioural & social sciences [ ] Ecological, evolutionary & environmental sciences

For a reference copy of the document with all sections, see nature.com/documents/nr-reporting-summary-flat.pdf

Life sciences study design

All studies must disclose on these points even when the disclosure is negative.

Sample size	No statistical methods were used to predetermine the sample size. Required sample sizes were estimated based on our experience performing similar experiments in previous publications.
Data exclusions	No data were excluded.
Replication	Consistent results obtained from at least three biological replicates with more than two technical replicates per experiment were used in the manuscript.
Randomization	Animals were randomly assigned to either experimental or control groups.
Blinding	Investigators were not blinded to the human and animal groups for experiments and when analyzing protein aggregates in immunofluorescence analysis.

Reporting for specific materials, systems and methods

We require information from authors about some types of materials, experimental systems and methods used in many studies. Here, indicate whether each material, system or method listed is relevant to your study. If you are not sure if a list item applies to your research, read the appropriate section before selecting a response.

Materials & experimental systems

n/a	Involved in the study	
[ ]	[x]	Antibodies
[ ]	[x]	Eukaryotic cell lines
[x]	[ ]	Palaeontology
[ ]	[x]	Animals and other organisms
[x]	[ ]	Human research participants
[x]	[ ]	Clinical data

Methods

n/a	Involved in the study	
[x]	[ ]	ChIP-seq
[x]	[ ]	Flow cytometry
[x]	[ ]	MRI-based neuroimaging

Antibodies

Antibodies used

Tau-13 BioLegend # MMS-520R (IF/PLA: 1:200, WB: 1:10,0000)
TOMA-2 In-house Tau Oligomeric Monoclonal Antibody 2 (IF/PLA: 1:200)
TTC35 In-house Toxic Tau Conformers (IP: 75 ug)
T22 In-house Tau Oligomer (IF: 1:300)
Musashi1 Santa-Cruz SC-135721 (IP: 2 ug)
Musashi1 [EP1302] Abcam ab52865 (IF/PLA: 1:250, WB: 1:1000)
Musashi2 [EP1305Y] Abcam ab76148 (IF/PLA: 1 ug/mL, WB: 1:1000)
GAPDH Abcam ab9485 (WB: 1:1000)
LaminA Abcam ab26300 (IF: 1 ug/mL, WB 1:1000)

	LaminB1 [EPR8985(B)] Abcam ab133741 (WB: 1:1000)<br>Histone3 [EPR17785] Abcam ab201456 (WB: 1:1000)<br>Alexa Fluor Anti-Mouse 488 Invitrogen A11029 (IF: 1:200)<br>Alexa Fluor Anti-Rabbit 488 Invitrogen A11034 (IF: 1:200)<br>Alexa Fluor Anti-Rabbit 568 Invitrogen A11036 (IF: 1:200)<br>Alexa Fluor Anti-Mouse 568 Invitrogen A11031 (IF: 1:200)<br>GE Healthcare HRP-conjugated anti-rabbit IgG (WB: 1:6000)<br>GE Healthcare HRP-conjugated anti-mouse IgG (WB: 1:6000)
Validation	All antibodies above are in everyday use or have been validated in other literature for use in cell, mice and human brain tissues. We also confirmed that each antibody for immunofluorescence stained in a typical cellular pattern and brain-wide distributions at its target proteins. We provide detailed catalog number, source, and dilution for each antibody used in the current study.

Eukaryotic cell lines

Policy information about cell lines

Cell line source(s)	HEK-293, iHEK WT-tau, iHEK P301L-tau (Dr. Laura J. Blair, University of South Florida Tampa, FL laurablair@usf.edu)
Authentication	Cells were authenticated by the supplier.
Mycoplasma contamination	All cell lines tested negative for mycoplasma.
Commonly misidentified lines<br>(See ICLAC register)	N/A

Animals and other organisms

Policy information about studies involving animals; ARRIVE guidelines recommended for reporting animal research

Laboratory animals	Transgenic mice and their control littermates were obtained from breeding colonies, maintained at University of Texas Medical Branch (UTMB) Animal Facility. All offspring were genotyped using tail DNA (Gene Script). KO tau mice (TAU KO- B6.129X1-Mapttm1Hnd/J The Jackson Laboratory Stock Number 007251) did not express tau. Tg(Prnp-MAPT*P301L)JNPL3Hlmc Taconic Stock Number 2508. mThy1-hSNCA, line 15 - C57BL/6N-Tg(Thy1-SNCA)15Mjff/J The Jackson Laboratory Stock Number 017682.<br>Animal age information is included in materials and methods section.
Wild animals	No wild animals used.
Field-collected samples	No field-collected samples used.
Ethics oversight	University of Texas Medical Branch Animal Care and Use Committee approved the study protocols used. IACUC Protocol numbers: 1001002C, 0802013C.

Note that full information on the approval of the study protocol must also be provided in the manuscript.
